# Personalized treatment strategy for atypical endometrial hyperplasia with regards to age, comorbidities and endometrial receptor status

**DOI:** 10.1186/1878-5085-5-S1-A40

**Published:** 2014-02-11

**Authors:** Vadym M Goncharenko, Vasyl A Beniuk, Yaroslav M Vyniarskyi, Sergiy M Bashynskyi, Rostyslav V Bubnov

**Affiliations:** 1 Clinical hospital "Pheophania", Kyiv, Ukraine; 2 Bogomolets National Medical University, Third Department of Obstetrics and Gynecology, Kyiv, Ukraine; 3 Institute of Pathology, Helios Klinik, Berlin, Germany

## Introduction

Endometrial hyperplasia (EH) is a basis for malignant transformation, atypical hyperplasia (AEH) has the highest cancer threat. Existing methods as cryosurgery, laser-, and electrodestruction, thermoablation may lead to irrevocable destruction of the endometrium. After hormonal treatment EH relapses occur in 15, 9-27, 2%, due to the morphological heterogeneity of endometrial proliferation. The sensitivity to therapy and prognosis is largely determined by receptor status.

## The aim

was to develop treatment algorithm for atypical endometrial hyperplasia in reproductive age women with regard to age, comorbidities and endometrial receptor status.

## Materials and methods

We included 62 women aged 20 to 52 years (mean of 43,2 ± 2,3 years), treated in Clinical Hospital "Pheophania" with a diagnosis of AEH combined with dysfunctional uterine bleeding 52 (82.3%), inflammatory diseases of genitals - 48 (77.4%), endocrine diseases (obesity, thyroid disease, diabetes) - 22 (35.5%), which with sensitivity of maintaining reproductive to hormone therapy functions to were considered for personalized treatment. We performed hysteroresectoscopy using «Karl Storz» equipment, hormone therapy (GnRH agonists, progestins). All patients underwent clinical examination, transvaginal ultrasound scanning including sonoelastography and 3D/4D technology at 1, 3, 6 and 12 months after hysteroresectoscopy; histological study to determine the endometrium receptor applying immunohistochemical method. After 6 months, we performed control hysteroscopy with endometrial biopsy to assess therapy effectiveness. 22 women, who underwent hysteroscopy for infertility before the cycles of in vitro fertilization (IVF) were the control group.

## Results

Estradiol receptors were found in 65.2% of epithelial cells and in 42.6% of stromal (43.3% and 29.6% respectively in controls); progesterone receptors - in 44.3% of epithelial cells (52.4% in controls). For patients with low expression of progesterone receptor we performed personalized therapy considering age, comorbidities. In increased progestin receptors, we administered GnRH agonists (Diferelin) for 6 months, followed by recovery of menstrual function and purpose of progestins (observations 3 - 4.8%). Treatment was effective in 28 reproductive age women (45.1%); we observed normalisation of menstrual function and ultrasound characteristics of endometrium. In 16 women (25.8%), older than 35 years ablative hysteroresectoscopy was performed to eliminate atypical and basal layers in single block. In 3 women (4.8%) we observed EH relapse (polyposis) during progestins therapy, which required rehysteroresectoscopy, followed by appointment of GnRH agonists (Diferelin) for 6 months and progestins (endometrin). In our opinion, the cause of EH relapse was insufficient electrodestruction due to the specific uterus anatomy. In 14 patients (22.5%) with AEH and comorbidities (large uterine fibroids, ovarian cystadenoma) hystero/oophorectomies were performed.

## Conclusions

1. The study of hormone receptor status in patients with EH allows you to clearly define the treatment policy, avoid relapse, optimizing treatment and observation of such patients.

2. Personalized pharmacotherapy combined with conserving interventions enabled to treat patients with AEH and significantly reduce surgery and time of treatment.

3. Performing hysteroresectoscopy and subsequent hormone therapy is an effective treatment of recurrent AEH.

## Outlook and expert recommendations

It is recommended to promote programs for introduction of personalized outpatient gynecological care of new level of efficiency and patient safety.

